# A role for primary cilia in glutamatergic synaptic integration of adult-born neurons

**DOI:** 10.1186/2046-2530-1-S1-P31

**Published:** 2012-11-16

**Authors:** N Kumamoto, Y Gu, J Wang, S Jenoschka, K Takemaru, J Levine, S Ge

**Affiliations:** 1 Osaka University Graduate School of Medicine, Japan; 2 SUNY at Stony Brook, USA

## 

New neurons are continually born throughout adulthood in the subventricular zone of the lateral ventricle and in the subgranular zone of the dentate gyrus in the hippocampus. The sequential synaptic integration of adult-born neurons has been widely examined in rodents, but the mechanisms regulating the integration remain largely unknown. The primary cilium, a microtubule–based signaling center, plays essential roles in vertebrate development, including the development of the central nervous system. We examined the assembly and function of the primary cilium in the synaptic integration of adult-born hippocampal neurons. Strikingly, primary cilia are absent in young adult-born neurons but assemble precisely at the stage when newborn neurons approach their final destination, further extend dendrites and form synapses with entorhinal cortical projections. Conditional cilia deletion from adult-born neurons induced severe defects in dendritic refinement and synapse formation. Primary cilia deletion leads to enhanced Wnt/beta-catenin signaling which may account for these developmental defects. Taken together, our study identifies the assembly of primary cilia as a critical regulatory event in the dendritic refinement and synaptic integration of adult-born neurons.

